# Investigation of microcirculation in patients with venoarterial extracorporeal membrane oxygenation life support

**DOI:** 10.1186/s13054-018-2081-2

**Published:** 2018-08-19

**Authors:** Yu-Chang Yeh, Chen-Tse Lee, Chih-Hsien Wang, Yu-Kang Tu, Chien-Heng Lai, Yin-Chin Wang, Anne Chao, Chi-Hsiang Huang, Ya-Jung Cheng, Yih-Sharng Chen, on behalf of the NTUH Center of Microcirculation Medical Research (NCMMR)

**Affiliations:** 1 Department of Anesthesiology, National Taiwan University Hospital, College of Medicine, National Taiwan University, No 7, Chung Shang South Road, Taipei, Taiwan; 2 Department of Surgery, National Taiwan University Hospital, College of Medicine, National Taiwan University, No 7, Chung Shang South Road, Taipei, Taiwan; 3 Department of Public Health, Institute of Epidemiology & Preventive Medicine, National Taiwan University, No. 17, Xu-Zhou Road, Taipei, Taiwan

**Keywords:** Cardiogenic shock, Extracorporeal membrane oxygenation, Microcirculation, Survival

## Abstract

**Background:**

Microcirculatory dysfunction develops in both septic and cardiogenic shock patients, and it is associated with poor prognosis in patients with septic shock. Information on the association between microcirculatory dysfunction and prognosis in cardiogenic shock patients with venoarterial extracorporeal membrane oxygenation (VA-ECMO) support is limited.

**Methods:**

Sublingual microcirculation images were recorded using an incident dark-field video microscope at the following time points: within 12 h (T1), 24 h (T2), 48 h (T3), 72 h (T4), and 96 h (T5) after VA-ECMO placement. If a patient could be weaned off VA-ECMO, sublingual microcirculation images were recorded before and after VA-ECMO removal. Microcirculatory parameters were compared between 28-day nonsurvivors and survivors with VA-ECMO support. In addition, the microcirculation and clinical parameters were assessed as prognostic tests of 28-day mortality, and patients were divided into three subgroups according to microcirculation parameters for survival analysis.

**Results:**

Forty-eight patients were enrolled in this study. At T1, the observed heart rate, mean arterial pressure, inotropic score and lactate level of 28-day nonsurvivors and survivors did not differ significantly, but the perfused small vessel density (PSVD) and proportion of perfused vessels (PPV) were lower in the 28-day nonsurvivors than in the survivors. The PSVD and PPV were slightly superior to lactate levels in predicting 28-day mortality (area under curve of 0.68, 0.70, and 0.62, respectively). The subgroup with the lowest PSVD (< 15 mm/mm^2^) and PPV (< 64%) values exhibited less favorable survival compared with the other two subgroups.

**Conclusions:**

Early microcirculatory parameters could be used to predict the survival of cardiogenic shock patients with VA-ECMO support.

**Trial registration:**

ClinicalTrials.gov, NCT02393274. Registered on 19 March 2015.

## Background

An extracorporeal membrane oxygenation (ECMO) life support system can provide both cardiac and respiratory support to patients with heart failure, respiratory failure, or both [1–4]. It can afford time for the failed organs to recover or for the patients to receive further management. However, many patients can possibly die despite ECMO support. One of the key factors is whether the blood flow provided by the ECMO system can restore organ perfusion. The adequacy of macrocirculation may be determined by arterial pressure and the minute blood flow of venoarterial ECMO (VA-ECMO). However, the adequacy of the microcirculation remains a major unresolved clinical concern in patients with ECMO. Microcirculatory dysfunction has been observed in patients who have suffered septic shock or cardiogenic shock, and in patients who have undergone surgery [5–9]. Moreover, microcirculatory dysfunction is associated with poor prognosis in patients with severe sepsis and in patients who have suffered an out-of-hospital cardiac arrest [10–12]. Because information regarding the association between microcirculatory dysfunction and prognosis in patients with VA-ECMO support is limited [13], this study focused on comparing perfused small vessel density (PSVD) between 28-day nonsurvivors and survivors by visualizing their microcirculation using a third-generation video microscope within 12 h after VA-ECMO placement [14, 15]. In addition, the microcirculation and clinical parameters were assessed as prognostic tests of 28-day mortality, and the patients were divided into three subgroups according to microcirculation parameters for survival analysis.

## Methods

### Patients

This prospective observational study was approved by the Research Ethics Committee of National Taiwan University Hospital (approval number 201412045RINA) and registered on the ClinicalTrials.gov protocol registration system (NCT02393274). This study was conducted at National Taiwan University Hospital between June 2015 and August 2016. Participants for the study were selected from patients receiving ECMO support; they were screened and evaluated for eligibility within 12 h after ECMO placement. The inclusion criterion was that patients should have suffered from cardiogenic shock and received VA-ECMO support. Patients who were aged < 20 or > 80 years, those for whom sublingual microcirculation could not be measured within 12 h after VA-ECMO placement (i.e., placement occurred in the evening, during holidays, or when the research assistant was on leave), and those who were non-native speakers were excluded. Informed consent of the patients was obtained from their legally authorized representatives before enrollment in the study. Sublingual microcirculation images were recorded using an incident dark-field video microscope (CytoCam, Braedius Medical, Huizen, the Netherlands) [16]. The images were recorded at the following time points: within 12 h (T1), 24 h (T2), 48 h (T3), 72 h (T4), and 96 h (T5) after VA-ECMO placement. If the patients could be weaned off VA-ECMO support, sublingual microcirculation images were recorded at the following time points: before removal (R0), and 6 h (R1), 24 h (R2), 48 h (R3), and 72 h (R4) after VA-ECMO removal.

### VA-ECMO components and placement

For all enrolled patients, the VA-ECMO was placed in the femoral artery and vein using the cut-down method. The principal component of VA-ECMO included a heparin-bonded surface circuit, a centrifugal pump (BPX-80 Bio-Pump Plus, Medtronic, Anaheim, CA, USA), an oxygenator (Affinity NT, Medtronic), an oxygen-air blender (Model 3500 CP-G gas mixer, Sechrist, Anaheim, CA, USA), and a cannula (BE-HLS, Maquet, Turkey). To avoid possible malperfusion of the distal limb, an antegrade distal perfusion catheter was used when the mean pressure of the superficial femoral artery was below 50 mmHg [17]. All patients received standard management of VA-ECMO and routine intensive care unit (ICU) care. Data pertaining to the following parameters were recorded: age, gender, height, body weight, Acute Physiology and Chronic Health Evaluation (APACHE) II score, Sequential Organ Failure Assessment (SOFA) score [18], indications of VA-ECMO, VA-ECMO blood flow, heart rate, mean arterial pressure (MAP), lactate level, activated clotting time, hemoglobin, fluid balance, and inotropic score. The inotropic score was calculated as 100 × epinephrine dose (μg/kg/min) + 100 × norepinephrine dose (μg/kg/min) + dopamine dose (μg/kg/min) + dobutamine dose (μg/kg/min) [19]. The use of intra-aortic balloon pump or continuous arteriovenous hemofiltration was recorded if simultaneously used with VA-ECMO support. The length of VA-ECMO support, length of ICU and hospital stay, and survival status at 28 days were also recorded. Heparin was continuously infused to maintain an activated clotting time of 160–180 s if no active bleeding or other complications were observed.

### Measurements of sublingual microcirculation

At each time point, five video sequences (time length 6 s) were recorded from different sublingual sites and were digitally stored with code numbers to ensure blinding of patient information. Subsequent offline analyses were performed by a single observer who was blinded to the patient information. The most appropriate three sequences were selected for analysis using the semi-automated analysis software package Automated Vascular Analysis (AVA) 3.0 (Academic Medical Center, University of Amsterdam, Amsterdam, the Netherlands). According to the suggestions of a previously held roundtable conference for evaluating the microcirculation [20], the following parameters were investigated: a) total small vessel (less than 20 μm) density (TSVD); b) perfused small vessel density (PSVD); c) proportion of perfused vessels (PPV); d) microvascular flow index (MFI) score; and e) heterogeneity index (HI). The TSVD was automatically calculated by the software. The blood flow in small vessels was semiquantitatively classified using an ordinal scale of 0–3 in accordance with the methods described in our previous study [5]. Small vessels with a blood flow classification of 2 or 3 were considered perfused vessels, and the PSVD was automatically calculated by the software. The MFI score and HI were semiquantitatively calculated according to the suggestions of the roundtable conference [20]. The primary endpoint was determining the difference between PSVD of 28-day survivors and nonsurvivors at T1. Based on our experience, 20 patients per group is sufficient to detect a 17.5% difference of PSVD between the two groups, with an α level of 0.05 (two-tailed) and a β level of 0.2, assuming a control mean PSVD of 20 mm/mm^3^ with a standard deviation of 4.

### Prognostic tests of 28-day mortality and subgroup survival analysis

Receiver operating characteristic (ROC) curves and the corresponding area under the curve (AUC) were used for assessing the discriminative abilities of APACHE II score, lactate level, PSVD, and PPV at T1 for 28-day mortality. Cutoff points were calculated by obtaining the optimal Youden index (sensitivity + specificity – 1). Moreover, patients were divided into three groups according to the 25th and 75th percentiles of PSVD and PPV values for the 28-day survival analysis among the three groups.

### Statistical analysis

Data were analyzed using the statistical software SPSS 20 (IBM, Armonk, NY, USA). Normally distributed numerical data are expressed as mean (standard deviation), and data for 28-day survivors and nonsurvivors were compared using *t* tests. Non-normally distributed numerical data and the MFI score are expressed as median (interquartile range), and data for 28-day survivors and nonsurvivors were compared using the Mann–Whitney test. Categorical variables are described as a percentage and were compared using the chi-square test or Fisher’s exact test, as appropriate. A *p* value < 0.05 was considered significant.

## Results

### Patient characteristics

**Fig. 1:**
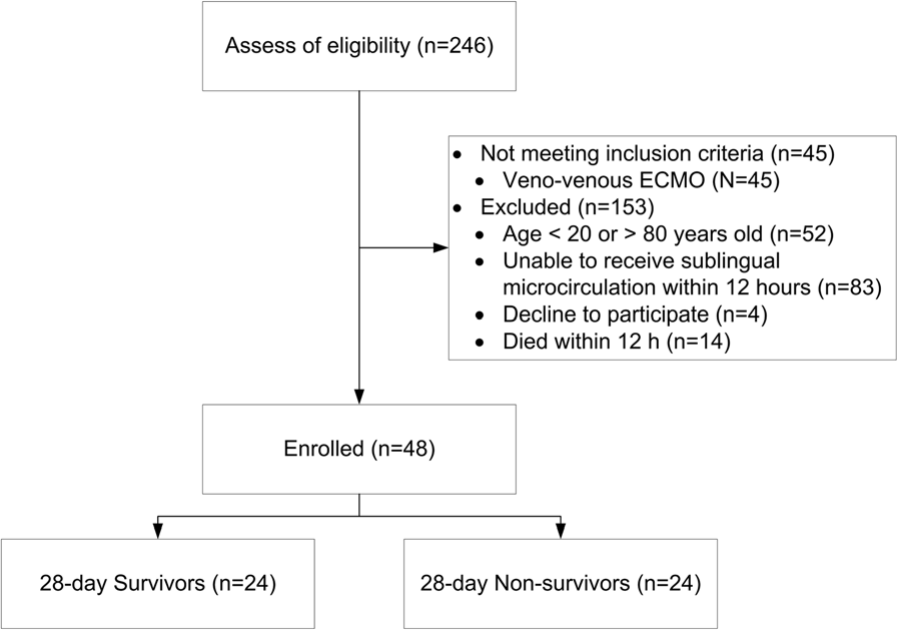
Consort flow chart of enrollment characteristics of patients receiving venoarterial extracorporeal membrane oxygenation life support (VA-ECMO). ECMO extracorporeal membrane oxygenation

A total of 246 patients receiving VA-ECMO support were screened for determining their eligibility for this trial. In total, 45 patients receiving venovenous ECMO did not meet the inclusion criterion, and 153 patients were excluded (Fig. 1). Therefore, 48 patients were enrolled in this study, and the 28-day survival rate was 50%. Values for the baseline characteristics, indications of VA-ECMO, APACHE II score, SOFA score, fluid balance, use of intra-aortic balloon pump or continuous arteriovenous hemofiltration, number of patients who underwent heart transplantation, number of discharged patients from 28-day survivors, length of ICU stay, and length of hospital stay are presented in Table 1.

**Table 1:** Patient characteristics

Characteristics	28-day survivors (*n* = 24)	28-day nonsurvivors (*n* = 24)	*p*
Age (years)	53 (13)	60 (12)	0.050
Female/male	6/18	7/17	0.745
Body weight (kg)	69 (17)	68 (14)	0.773
Height (cm)	168 (10)	161 (22)	0.272
Indication of ECMO			
Heart failure	12	10	
Postcardiotomy	1	1	
ECPR	10	11	
Septic shock	0	2	
ARDS with shock	1	0	
APACHE II_T1	19 (7)	26 (8)	0.002
SOFA_T1	13 (3)	14 (3)	0.208
APACHE II_T2	17 (7)	24 (9)	0.005
SOFA_T2	13 (4)	15 (3)	0.049
APACHE II_R0	15 (6)	20 (6)	0.014
SOFA_R0	11 (3)	16 (3)	< 0.001
Fluid balance 6 h (ml)	1019 (2016)	2801 (3210)	0.026
Fluid balance 24 h (ml)	266 (1221)	1955 (3027)	0.022
Fluid balance 48 h (ml)	−44 (922)	953 (2184)	0.104
IABP_T1, *n* (%)	8 (33%)	6 (25%)	0.752
CAVH_T1, *n* (%)	6 (25%)	12 (50%)	0.074
CAVH_T1–T5, *n* (%)	12 (50%)	18 (75%)	0.074
Heart transplant, *n* (%)	3 (13%)	1 (4%)	0.609
Length of ECMO support (day)	5 (3–8.5 [1–54])		
Survive to discharge	*n* = 22		
ICU stay (day)	18 (8–41 [4–70])		
Hospital stay (day)	39 (22–70 [11–127])		

Data are presented as mean (standard deviation), *n* (%), or median (interquartile range [range])

T1, T2, and T5 represent within 12, 24, and 96 h, respectively, after placement of extracorporeal membrane oxygenation life support system (ECMO)

R0 represents before removal of ECMO

*APACHE* Acute Physiology and Chronic Health Evaluation, *ARDS* acute respiratory distress syndrome, *CAVH* continuous arteriovenous hemofiltration, *ECPR* extracorporeal cardiopulmonary resuscitation, *IABP* intra-aortic balloon pump, *ICU* intensive care unit, *SOFA* Sequential Organ Failure Assessment

### Hemodynamic parameters, inotropic score, lactate level, and microcirculatory parameters at different time points

**Fig. 2:**
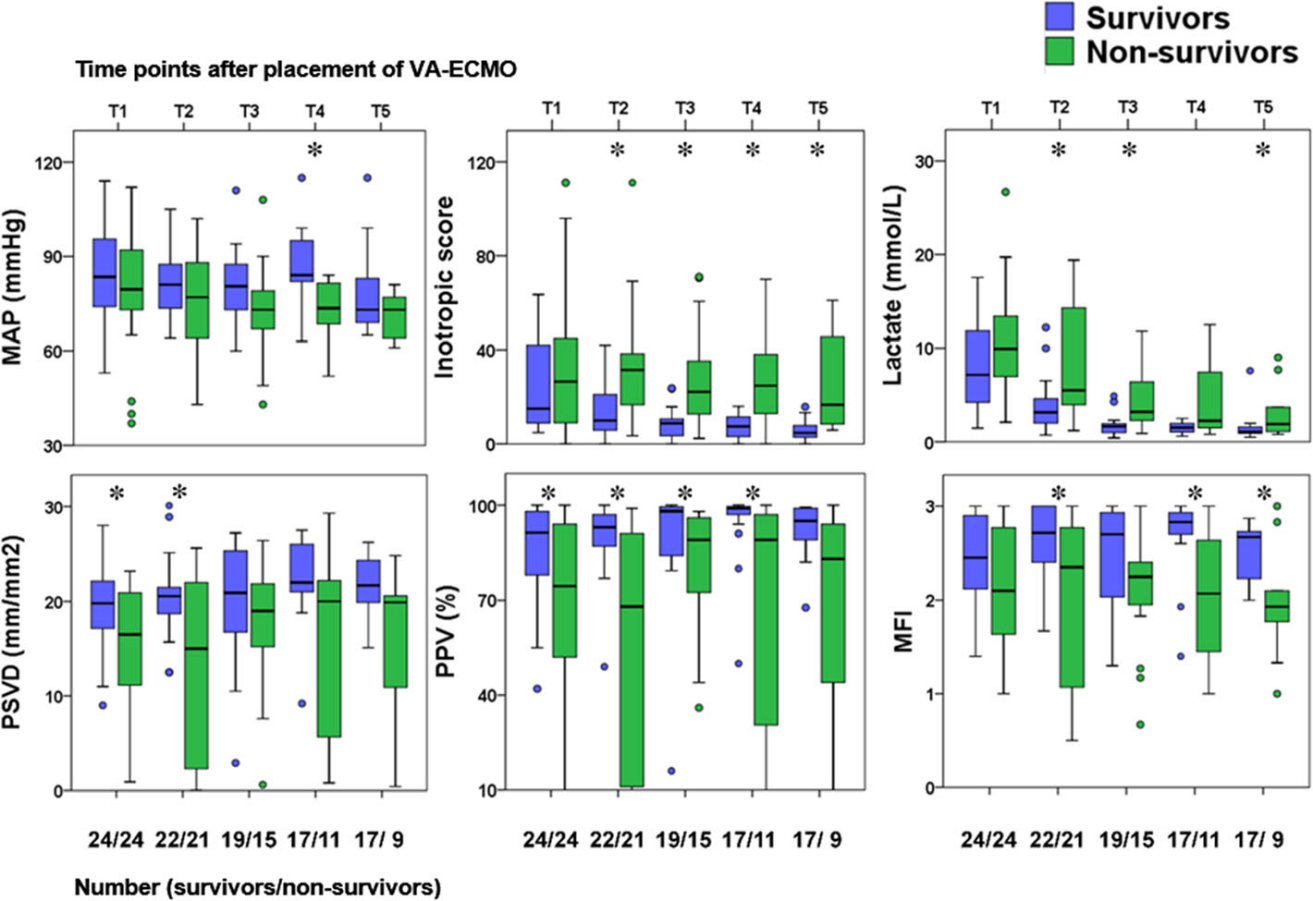
Mean arterial pressure (MAP), inotropic score, lactate level, and microcirculation parameters of 28-day survivors and nonsurvivors after placement of venoarterial extracorporeal membrane oxygenation life support (VA-ECMO). The time points after placement of VA-ECOM are presented as T1 (within 12 h), T2 (24 h), T3 (48 h), T4 (72 h), and T5 (96 h). *p < 0.05 between 28-day survivors and nonsurvivors. MFI microvascular flow index, PPV proportion of perfused vessels, PSVD perfused small vessel density

**Fig. 3:**
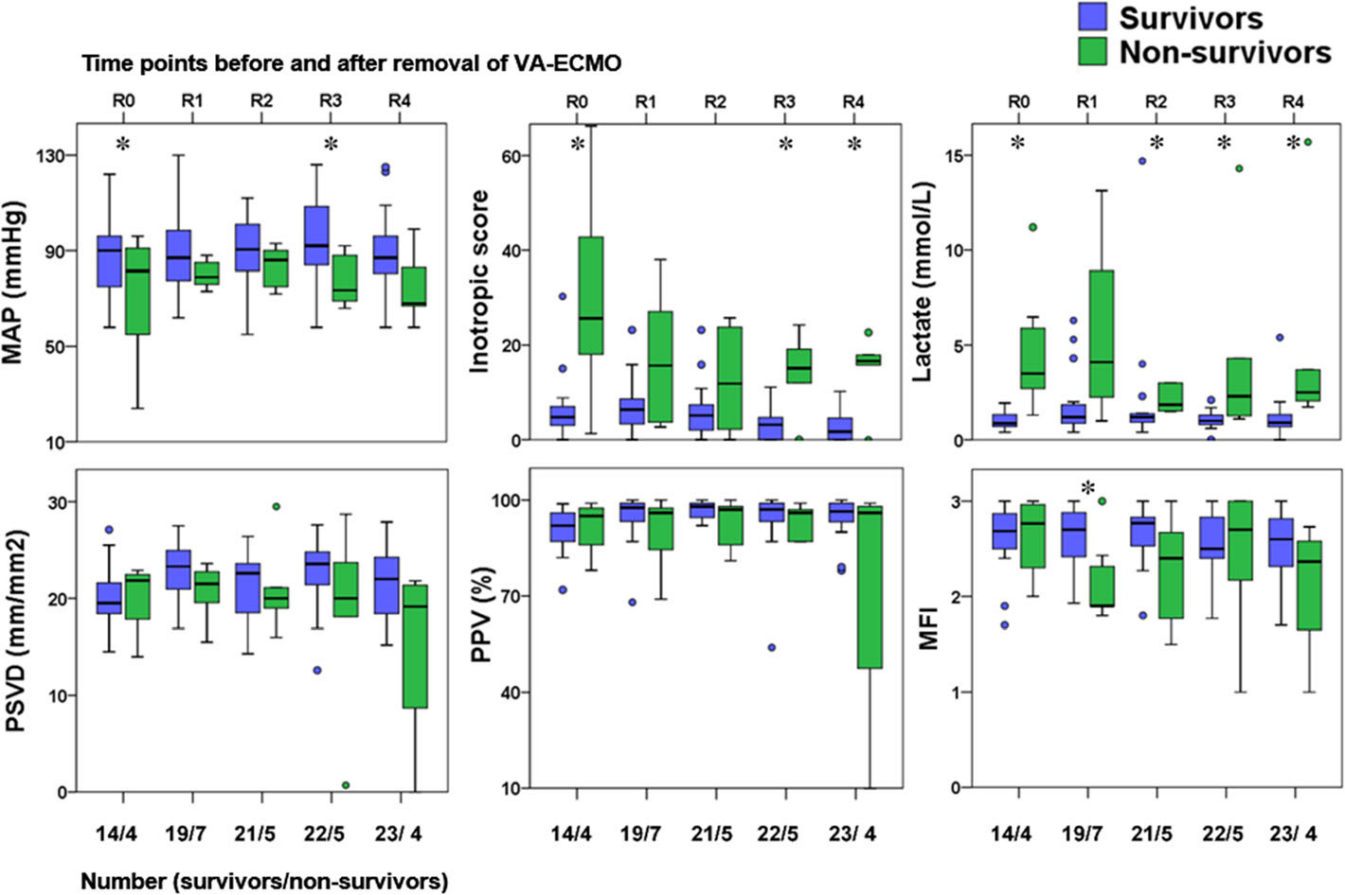
Mean arterial pressure (MAP), inotropic score, lactate level, and microcirculation parameters of 28-day survivors and nonsurvivors before and after removal of venoarterial extracorporeal membrane oxygenation life support (VA-ECMO). The time point before removal of VA-ECOM is presented as R0, and the time points after removal of VA-ECOM are presented as R1 (6 h), R2 (24 h), R3 (48 h), and R4 (72 h). *p < 0.05 between 28-day survivors and nonsurvivors. MFI microvascular flow index, PPV proportion of perfused vessels, PSVD perfused small vessel density

Values for the hemodynamic parameters, inotropic score, lactate level, and microcirculatory parameters at T1, T2, T3, T4, and T5 are shown in Fig. 2 and Table 2. At T1, the observed MAP, inotropic score, and lactate level did not differ significantly between the 28-day nonsurvivors and survivors, but the PSVD and PPV for the 28-day nonsurvivors were lower than those for the survivors. Values for the hemodynamic parameters, inotropic score, lactate level, and microcirculatory parameters at R0, R1, R2, R3, and R4 are presented in Fig. 3. At R0, the MFI score did not differ between the 28-day nonsurvivors and survivors; by contrast, at R1, the MFI score for the 28-day nonsurvivors was lower than that for the survivors. .

**Table 2:** ECMO blood flow and microcirculatory parameters of 28-day survivors and nonsurvivors at T1, T2, T3, T4, and T5

Parameters	28-day survivors	28-day nonsurvivors	*p*
T1 (within 12 h after ECMO placement)	(*n* = 24)	(*n* = 24)	
ECMO blood flow (L/min)	2.3 (0.8)	2.5 (0.6)	0.191
TSVD (mm/mm^2^)	22.5 (2.7)	22.0 (3.5)	0.576
HI	0.30 (0.1–0.44)	0.38 (0.09–0.71)	0.909
T2 (24 h after ECMO placement)	(*n* = 22)	(*n* = 21)	
ECMO blood flow (L/min)	2.2 (0.4)	2.5 (0.5)	0.022
TSVD (mm/mm^2^)	22.8 (3.6)	22.8 (3.4)	0.994
HI	0.21 (0–0.41)	0.24 (0.18–0.57)	0.269
T3 (48 h after ECMO placement)	(*n* = 19)	(*n* = 15)	
ECMO blood flow (L/min)	2.1 (0.4)	2.5 (0.9)	0.176
TSVD (mm/mm^2^)	21.7 (4.4)	22.7 (3.4)	0.442
HI	0.2 (0.07–0.3)	0.33 (0.14–0.44)	0.040
T4 (72 h after ECMO placement)	(*n* = 17)	(*n* = 11)	
ECMO blood flow (L/min)	2.2 (0.4)	2.8 (1.2)	0.097
TSVD (mm/mm^2^)	23.8 (3.4)	21.8 (4.5)	0.177
HI	0.14 (0.05–0.25)	0.18 (0.05–0.32)	0.353
T5 (96 h after ECMO placement)	(*n* = 17)	(*n* = 9)	
ECMO blood flow (L/min)	2.1 (0.6)	3.0 (1.4)	0.089
TSVD (mm/mm^2^)	23.3 (2.9)	21.3 (4.8)	0.183
HI	0.31 (0.1–0.54)	0.18 (0–0.56)	0.458

Data are presented as mean (SD) or median (interquartile range)

*ECMO* extracorporeal membrane oxygenation life support system, *HI* heterogeneity index, *TSVD* total small vessel density

### Prognostic tests and subgroup survival analysis of 28-day mortality

**Fig. 4:**
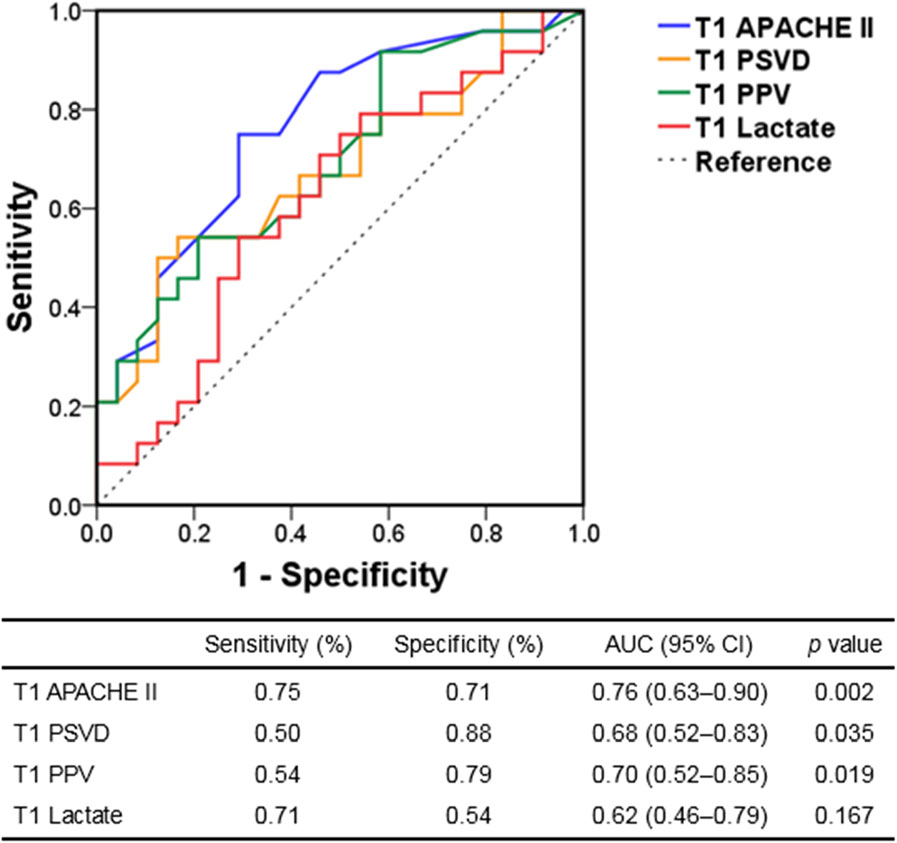
Prognostic tests of 28-day mortality. APACHE Acute Physiology and Chronic Health Evaluation, AUC area under the curve, CI confidence interval, PPV proportion of perfused vessels, PSVD perfused small vessel density

**Fig. 5:**
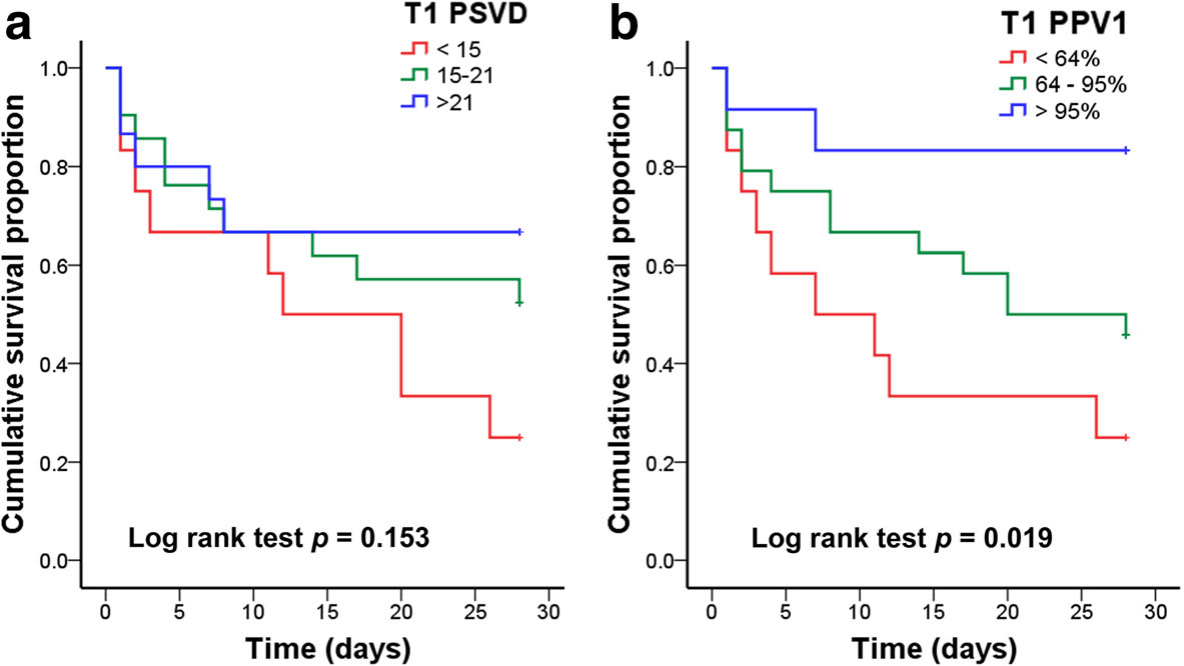
Twenty-eight-day survival curves based on subgrouping by perfused small vessel density (PSVD) and proportion of perfused vessels (PPV). Patients were divided into three subgroups according to the 25th and 75th percentiles of **a** PSVD (mm/mm^2^) and **b** PPV (%) values

The ROC curves for prognostic tests of 28-day mortality are illustrated in Fig. 4. According to the ROC curve analysis, the threshold values of the APACHE II score, lactate level, PSVD, and PPV were 22.5, 7.5 mmol/l, 16.2 mm/mm^2^, and 76.5%, respectively. The 28-day survival curves based on the subgrouping according to PSVD and PVD values at T1 are presented in Fig. 5. The patients in the two subgroups with higher PSVD and PPV values exhibited greater survival than those in the subgroup with the lowest PSVD and PPV values.

## Discussion

This prospective observational study showed that microcirculatory dysfunction was more severe in 28-day nonsurvivors than in survivors with VA-ECMO support. Moreover, this study revealed that the PSVD and PPV at T1 could be used to predict the survival of such patients. Furthermore, when the patients were divided into three subgroups according to the 25th and 75th percentiles of PSVD and PPV values at T1, the patients in the two subgroups with higher PSVD and PPV values exhibited greater survival than those in the subgroup with the lowest PSVD and PPV values.

The main finding of this study is that the PSVD and PPV at T1 in the 28-day nonsurvivors were lower than in the survivors, but the observed MAP, inotropic score, and lactate level at T1 did not differ significantly between the 28-day nonsurvivors and survivors. This disparity was consistent with the notion that microcirculatory dysfunction can occur in normal macrocirculation parameters [21, 22]. Therefore, we suggest that MAP might not be suitable as an optimal or final resuscitation goal for patients with cardiogenic shock receiving VA-ECMO support. However, measuring microcirculation parameters may help to predict outcomes and provide information on the adequacy of tissue perfusion. Further studies are required to investigate the effect of improving microcirculation on survival. In addition, fluid balance was higher in the 28-day nonsurvivors than in the survivors. Fluid overload might have resulted from the higher severity of shock in the 28-day nonsurvivors. Such overload of fluid might result in increased diffusive distance of the small vessels, reducing the ability of oxygen to reach the tissue cells [23].

Our result that microcirculatory dysfunction was more severe in 28-day nonsurvivors than in survivors with VA-ECMO support agrees with the findings of Kara et al. [13]. However, there are several differences between the findings of the two studies. First, we found that the APACHE II score for the 28-day nonsurvivors was higher than that for the survivors. However, in the study by Kara et al., the APACHE II scores for 28-day nonsurvivors and survivors did not differ significantly. Second, the PPV value for both 28-day survivors and nonsurvivors in our study are lower than those reported by Kara et al. Third, in our study, the level of ECMO blood flow at T2 was higher in the 28-day nonsurvivors than in the survivors. However, in the study of Kara et al., the levels of ECMO blood flow in survivors and nonsurvivors did not differ significantly. In addition, the level of VA-ECMO blood flow in the study by Kara et al. is higher than that in our study. There are several explanations for the different findings of the two studies. First, we enrolled more patients in this study than did Kara et al. (48 vs 24 patients). Second, we measured baseline microcirculatory parameters within the first 12 h after VA-ECMO placement, whereas Kara et al. measured these parameters within the first 24 h. Third, the two studies had different definitions of diameter of small vessels (< 20 μm vs < 25 μm). In addition, Kara et al. used the PSVD of all vessels (< 100 μm) to predict survival from ROC curves. Fourth, the two studies had different definitions of mortality (28-day mortality vs ICU mortality).

In our study, the PSVD derived for the 28-day survivors at R4 was still lower than that derived for 70 healthy volunteers in our unpublished study (21.8 (3.7) vs. 25.2 (2.3) mm/mm^2^, *p* < 0.001). Persistent microcirculatory dysfunction in patients with VA-ECMO support perhaps results from primary diseases, inflammatory response of VA-ECMO [24], and hemolysis-associated residual endothelium dysfunction [25, 26]. Persistent microcirculatory dysfunction is associated with organ failure and death in patients with septic shock [22]. Additional studies are required to investigate the effect of persistent microcirculatory dysfunction on organ dysfunction in patients with VA-ECMO support. The MFI scores for the 28-day nonsurvivors and survivors did not differ significantly before VA-ECMO removal. However, within 6 h after VA-ECMO removal, the MFI score for the 28-day nonsurvivors became lower than that for the survivors. Additional studies are required to investigate changes in MFI score while decreasing VA-ECMO blood flow before VA-ECMO removal; this may provide information to predict the microcirculation status following VA-ECMO removal. Additional studies may also compare the predictabilities of microcirculatory parameters with the current VA-ECMO weaning predictors [1, 27, 28]. Moreover, lactate levels were higher in the 28-day nonsurvivors than in the survivors at R0, R2, R3, and R4. Thus, lactate levels might provide further information before and after the removal of VA-ECMO.

This study had several limitations. First, the mechanism of microcirculatory dysfunction and its effects on mortality might vary in different primary etiologies of cardiogenic shock, but the sample size of this study was too small to investigate such variances. Second, the number of 28-day nonsurvivors decreased at other time points due to death after T1. Comparisons between the variables of 28-day nonsurvivors and survivors at other time points might not have had sufficient power to detect significant differences. In addition, this meant the trial was not suitable for a nonparametric analysis of variance (ANOVA) for repeated measures. These data provide preliminary information for further studies to investigate the microcirculation at other time points after VA-ECMO placement and VA-ECMO removal. Third, the optimal cutoff points of the microcirculatory parameters could have been influenced by the defined diameter of small vessels (< 20 μm or < 25 μm) or the range of observed microcirculatory vessels (only small vessels or total vessels). We suggest that the different primary etiologies of cardiogenic shock might affect different types of microcirculatory vessels. Additional studies are required to investigate the optimal defined diameter of small vessels and the optimal range of observed microcirculatory vessels in different etiologies of cardiogenic shock.

## Conclusions

We show that early microcirculatory parameters could be used to predict the survival of patients with cardiogenic shock with VA-ECMO support. Additional studies are required to investigate whether improving microcirculation can improve the survival of patients with cardiogenic shock receiving VA-ECMO support.

## Abbreviations

ANOVA: Analysis of variance
APACHE: Acute Physiology and Chronic Health Evaluation
AUC: Area under the curve
AVA: Automated Vascular Analysis
ECMO: Extracorporeal membrane oxygenation
HI: Heterogeneity index
ICU: Intensive care unit
MAP: Mean arterial pressure
MFI: Microvascular flow index
PPV: Proportion of perfused vessels
PSVD: Perfused small vessel density
ROC: Receiver operating characteristic
SOFA: Sequential Organ Failure Assessment
TSVD: Total small vessel density
VA-ECMO: Venoarterial extracorporeal membrane oxygenation
